# Molecular characterizations of antibiotic resistance, biofilm formation, and virulence determinants of *Pseudomonas aeruginosa* isolated from burn wound infection

**DOI:** 10.1002/jcla.24850

**Published:** 2023-02-17

**Authors:** Shirin Ghasemian, Morteza Karami‐Zarandi, Hamid Heidari, Saeed Khoshnood, Ebrahim Kouhsari, Sobhan Ghafourian, Abbas Maleki, Hossein Kazemian

**Affiliations:** ^1^ Department of Microbiology, Faculty of Medicine Ilam University of Medical Sciences Ilam Iran; ^2^ Department of Microbiology, Faculty of Medicine Zanjan University of Medical Sciences Zanjan Iran; ^3^ Department of Microbiology, Faculty of Medicine Shahid Sadoughi University of Medical Sciences Yazd Iran; ^4^ Clinical Microbiology Research Center Ilam University of Medical Sciences Ilam Iran; ^5^ Laboratory Sciences Research Center Golestan University of Medical Sciences Gorgan Iran; ^6^ Department of Laboratory Sciences, Faculty of Paramedicine Golestan University of Medical Sciences Gorgan Iran

**Keywords:** biofilm, burn injuries, carbapenem‐resistant, ERIC‐PCR, *Pseudomonas aeruginosa*

## Abstract

**Background:**

Burn injuries result in disruption of the skin barrier against opportunistic infections. *Pseudomonas aeruginosa* is one of the main infectious agents colonizing burn wounds and making severe infections. Biofilm production and other virulence factors along with antibiotic resistance limit appropriate treatment options and time.

**Materials and Methods:**

Wound samples were collected from hospitalized burn patients. *P. aeruginosa* isolates and related virulence factors identified by the standard biochemical and molecular methods. Antibiotic resistance patterns were determined by the disc diffusion method and β‐lactamase genes were detected by polymerase chain reaction (PCR) assay. To determine the genetic relatedness amongst the isolates, enterobacterial repetitive intergenic consensus (ERIC)‐PCR was also performed.

**Results:**

Forty *P. aeruginosa* isolates were identified. All of these isolates were biofilm producers. Carbapenem resistance was detected in 40% of the isolates, and *bla*_TEM_ (37/5%), *bla*_VIM_ (30%), and *bla*_CTX‐M_ (20%) were the most common β‐lactamase genes. The highest resistance was detected to cefotaxime, ceftazidime, meropenem, imipenem and piperacillin, and 16 (40%) isolates were resistant to these antibiotics. The minimum inhibitory concentrations (MIC) of colistin was lower than 2 μg/mL and no resistance was observed. Isolates were categorized to 17 MDR, 13 mono‐drug resistance, and 10 susceptible isolates. High genetic diversity was also observed among the isolates (28 ERIC types) and most carbapenem‐resistant isolates were classified into four main types.

**Conclusion:**

Antibiotic resistance, particularly carbapenem resistance was considerable among the *P. aeruginosa* isolates colonizing burn wounds. Combining carbapenem resistance with biofilm production and virulence factors would result in severe and difficult‐to‐treat infections.

## Graphical abstract

All of the isolates were biofilm producers. Carbapenem resistance was detected in 40% of the isolates, and *bla*_TEM_ (37/5%), *bla*_VIM_ (30%), and *bla*_CTX‐M_ (20%) were the most common β‐lactamase genes. The minimum inhibitory concentrations of colistin was lower than 2 μg/mL and no resistance was observed. Isolates were categorized to 17 MDR, 13 mono‐drug resistance, and 10 susceptible isolates. High genetic diversity was also observed among the isolates and most carbapenem‐resistant isolates were classified into four main ERIC types.

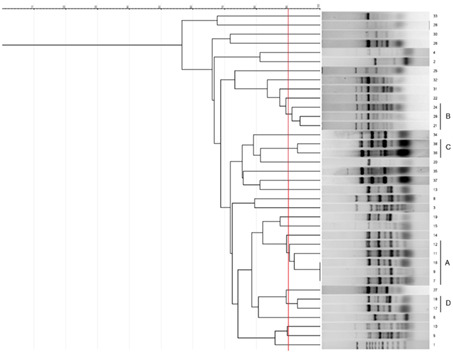

## 1 INTRODUCTION

Normal and intact skin is a barrier against infective agents, such as *Pseudomonas aeruginosa*.^1^ Burn injuries destroy the skin protection against infection and disrupt the physiologic function of the immune system, and burn patients are at high risk of acquiring hospital‐associated infections.^2^ *P. aeruginosa* is an aerobic Gram‐negative bacilli and accounts for opportunistic or nosocomial infections in burn patients, cystic fibrosis, and immunocompromised individuals.^3^

*Pseudomonas aeruginosa* possesses a wide range of virulence factors such as elastase, exoenzymes, and exotoxin A which are regulated by cell‐to‐cell signaling systems. The main virulence factor produced by isolates of *P. aeruginosa* is exotoxin A which has an important role in the pathogenesis of this microorganism.^4^ Also, flagella and pili have a key role as virulence factors independently.^5^ *P. aeruginosa* is able to enhance the excretion of virulence determinants in the cytoplasm of target cells through a type III secretion system. These factors are associated with higher mortality, especially in burn patients.^6^ Moreover, biofilm formation is a basic and critical virulence factor that improves bacterial survival in harsh circumstances such as dryness or the presence of antiseptics.^7^ Biofilm also is one of the main strategies for antibiotic resistance that increases horizontal gene transfer between susceptible and resistant strains.^8^ It is a complex aggregate of bacteria encased in alginate polysaccharides and encoded by the *algD* gene.^7^ Biofilm also makes a barrier between bacterial cells and antibiotics or immune responses.^8^ *P. aeruginosa* destroys natural structures of skin or mucous membranes using protease (such as elastase or Las), phospholipase (Plc), neuraminidase (Nan), and exotoxins. They are among those virulence factors that destroy connective tissue proteins, cytokines, cell membranes, and antibodies and modulate *P. aeruginosa* infections in proper sites such as burned skin or cystic fibrosis lungs.^9^ In burn injuries, the natural defense of skin is destructed, and exposed matrix proteins and inflammatory factors accelerate the colonization of *P. aeruginosa* and infection.^10^

Besides these virulence factors that make microorganism a destructive pathogen, antibiotic resistance also complicates the treatment of *P. aeruginosa* infections. Antibiotic resistance is mediated by various strategies such as β‐lactamases, efflux pumps and mutations, and multi‐drug‐resistant (MDR) isolates harbor several mechanisms for antibiotic resistance.^11^ In *P. aeruginosa* different β‐lactamases like extended spectrum β‐lactamases (ESBLs) and metallo‐β‐lactamases (MBLs) cause resistance to β‐lactam antibiotics.^11^ The combination of β‐lactamase‐producing phenotype and virulence factors creates a highly human pathogen, especially in burn patients.^10^

Characterization of local epidemiology and determination of genetic relatedness of the drug‐resistant isolates is necessary to control their dissemination in healthcare setting.^12^ To determine the genotypic relationship amongst *P. aeruginosa* isolates, various genotyping methods including, multilocus sequence typing (MLST) and pulsed‐field gel electrophoresis (PFGE) have been used.^13^ Furthermore, polymerase chain reaction (PCR)‐based techniques such as enterobacterial repetitive intergenic consensus (ERIC)‐PCR are rapid, cost‐effective, reproducible, and reliable typing methods with acceptable discriminatory power for non‐fermenting Gram‐negative bacilli.^13^, ^14^

In the current study, we aimed to assess virulence factors, biofilm formation ability, β‐lactamase associated genes, and the genetic relationship amongst *P. aeruginosa* isolates, obtained from in burn wound infections.

## 2 MATERIALS AND METHODS

### 2.1 Bacterial isolates

In this study, clinical isolates of *P. aeruginosa* were isolated between March 2020 and September 2020 from burn wound samples in the selected hospitals in Tehran, and Ahvaz, Iran. All patients or their legal guardians provided informed written consent, and this study was approved by the Ethics Committee of Ilam University of Medical Sciences (IR.MEDILAM.REC.1399.237). Samples were inoculated on blood agar and MacConkey agar mediums immediately and *P. aeruginosa* isolates were identified by conventional biochemical tests including, Gram stain, oxidase, catalase, oxidation‐fermentation (OF) test, and the Triple Sugar Iron Agar (TSI) tests.

### 2.2 Drug susceptibility tests

Antibiotic susceptibility test (DST) was performed for isolates by disc diffusion method according to Clinical Laboratory Standard Institute (CLSI) 2020 guideline.^15^ Imipenem (10 μg), meropenem (10 μg), cefotaxime (30 μg), ceftazidime (30 μg), piperacillin (100 μg), ciprofloxacin (5 μg) and gentamycin (10 μg) discs were used to determine the resistance pattern. In addition, the micro‐broth dilution method was applied to determine the susceptibility situation to colistin.

### 2.3 Phenotypic tests for ESBL, carbapenemase, and metallo β‐lactamase

All isolates were screened for the production of ESBL and MBLs enzymes using the combination disc method. In brief, an overnight incubated suspension of each isolate was inoculated on Muller‐Hinton agar media. Then, ceftazidime and ceftazidime/clavulanic acid discs were used to determine ESBL enzymes. Imipenem and EDTA discs were also used to detect MBLs enzymes. Carbapenemase activity was assessed using the carba‐NP test method, as described previously.^16^

### 2.4 Biofilm assay

Biofilm formation assay was performed as described previously.^17^ In brief, *P*. *aeruginosa* isolates were inoculated in 5 mL trypticase soy broth (TSB) and overnight incubated at 37°C. Then a concentration equal to 0.5 McFarland standard was prepared in TSB and each well of a flat‐bottomed polystyrene 96‐well microtiter plate was inoculated with 100 μL of these dilutions. After 24 h incubation at 37°C, the supernatant was removed and wells were rinsed with normal saline solution (0.9% NaCl). Adherent biofilms were fixed with 99% ethanol. The solutions were removed, and the plate was air‐dried, and stained with crystal violet (1.5%) for 20 min after that the unbound stain was rinsed with water. The dye was solubilized in 150 μL of 30% (v/v) acetic acid. The optical densities (OD) of the wells were measured by a microplate reader at 550 nm. The whole process was performed in triplicate for each isolate, and *P. aeruginosa* ATCC 27853 and sterile broth were used as a positive and negative control. A cut‐off value (ODc) was determined and it is defined as three standard deviations (SD) above the mean OD of the negative control: ODc = average OD of negative control + (3 × SD of negative control). The isolates were categorized into the four following groups based on the OD: non‐biofilm producer (OD < ODc); weak‐biofilm producer (ODc < OD <2 × ODc); moderate‐biofilm producer (2 × ODc < OD <4 × ODc); strong‐biofilm producer (4 × ODc < OD).^17^, ^18^

### 2.5 Molecular detection of virulence and resistance

The whole genomic DNA was extracted from pure colonies of isolated *P. aeruginosa* isolates using the boiling method. Briefly, a few colonies were dissolved in sterile distilled water and placed in a dry bath at 95°C for 15 min. Then the isolates were placed at −20°C for 10 min and then centrifuged at 13,000 rpm for 10 min. The supernatant was used as a DNA template. The extracted DNA was kept at −20°C until processed. The quality of the extracted DNA was determined using an absorbance ratio of 260/280 nm by a NanoDrop spectrophotometer. The genes encoding virulence factors (*algD*, *lasB*, *plcH*, *nan1*, *exoS*, and *exoA*) and β‐lactamase resistance genes (ESBL genes [*bla*_CTXM_ *, bla*_SHV_ *, bla*_TEM_] and carbapenemase genes [*bla*_VIM_ *, bla*_IMP_ *, bla*_NDM_ *, bla*_OXA‐48_ *, bla*_OXA‐23_, and *bla*_OXA‐11_]) were detected by PCR method using the specific primers (Table 1).^19^, ^20^, ^21^, ^22^ Then, 1% agarose gel electrophoresis and gel staining (stain load dye (CinnaGen Co, Iran)) were conducted for the analysis of PCR products.

**TABLE 1:** Primers were used for amplification of virulence and β‐lactamase genes.

Gene	Primer sequence	Amplicon size (bp)	Reference
*exoA*	F: GACAACGCCCTCAGCATCACCAGC R: CGCTGGCCCATTCGCTCCAGCGCT	396	^19^
*nan1*	F: ATG AAT ACT TAT TTT GAT AT R: CTA AAT CCA TGC TCT GAC CC	1316	^20^
*lasB*	F: AGCCATCACCGAAGTCAAGG R: CGGGAATCAGGTAGGAGACG	250	^21^
*ExoS*	F: CTT GAA GGG ACT CGA CAA GG R: TTC AGG TCC GCG TAG TGA AT	504	^21^
*algD*	F: ATG CGA ATC AGC ATC TTT GGT R: CTA CCA GCA GAT GCC CTC GGC	1310	^21^
*plcH*	F: GAA GCC ATG GGC TACTTCAA R: AGA GTG ACG AGG AGC GGTAG	307	^21^
*bla*_SHV_	F: GCCCGGGTTATTCTTATTTGTCGC R: TCTTTCCGATGCCGCCGCCAGTCA	1013	^22^
*bla*_TEM_	F: TCCGCTCATGAGACAATAACC R: ATAATACCGCACCACATAGCAG	300	^22^
*bla*_CTX‐M_	F: TTTGCGATGTGCAGTACCAGTAA R: CGATATCGTTGGTGGTGCCATA	455	^22^
*bla*_VIM_	F: GATGGTGTTTGGTCGCATA R: CGAATGCGCAGCACCAG	390	^22^
*bla*_IMP_	F: AGCCCATAGTTAACCCCGCC R: CTGGCTTAATTCTCAATCCATCCC	114	^22^
*bla*_NDM_	F: GGTTTGGCGATCTGGTTTTC R: CGGAATGGCTCATCACGATC	621	^22^
*bla*_oxa‐23_	F: TGGAAGGGCGAGAAAAGGTC R: TTGCCCAACCAGTCTTTCCA	400	^22^
*bla*_oxa‐48_	F: GCGTGGTTAAGGATGAACAC R: CATCAAGTTCAACCCAACCG	438	^22^
*bla*_oxa‐11_	F: CGAGTACGGCATTAGCTGGT R: CTCTTGGCTTTCCGTCCCAT	250	^22^

### 2.6 Enterobacterial repetitive intergenic consensus (ERIC‐PCR)

To characterize the genetic relatedness among the isolates, ERIC‐PCR was performed using followed primers, ERIC1 5′‐ATGTAAGCTCCTGGGGATTCAC‐3′ and ERIC2 5′‐AAGTAAGTGACTGGGGTGAGCG‐3′, as described previously.^13^ The PCR protocol consisted of a pre‐denaturation step at 95°C for 5 min, followed by 30 cycles of 60 s at 95°C, 50 s at 59°C, and 60 s at 72°C. A final extension step was done at 72°C for 10 min. PCR products were separated by electrophoresis in 1.5% agarose gels with 0.5× TBE (Tris/Boric acid/EDTA) buffer. DNA bands were visualized using UV light after staining with safe stain load dye. The GelJ software version 2.0 was used to analyze ERIC patterns^23^ and the isolates with a similarity coefficient ≥90% were clustered in the same genotypes. In other words, the isolates with equal or more than 90% similarity in their banding patterns were considered the same ERIC type.

### 2.7 Statistical analysis

The SPSS version 22.0 (SPSS, Inc.) was used to analyze the data. Pearson Chi‐Square test was used to determine the statistically significant correlation between the existence of genes and antibiotic resistance or biofilm production. In addition, *p*‐value <0.05 was considered as a significance level. The results are presented as descriptive statistics in terms of relative frequency.

## 3 RESULTS

### 3.1 Isolates and drug susceptibility

*Pseudomonas aeruginosa* isolates were identified by various tests that included: Gram‐negative bacilli, motile, oxidase and catalase positive, bluish green pigmentation, and glucose oxidizer. In this study, 40 *P. aeruginosa* isolates were collected from burn wound samples of 23 male and 17 female hospitalized patients. The mean age of the patients were 26 ± 5 years and 21 patients had Neck and face skin wound, 12 patients had hands and arms wound and 7 patients had full body wounds. The highest resistance was detected for cefotaxime, ceftazidime, meropenem, imipenem and piperacillin, and 16 (40%) isolates were resistant to these antibiotics. The resistance rate to ciprofloxacin and gentamicin was slightly lower and 12 (30%) isolates were resistant to them (Table 2). Minimum inhibitory concentrations (MIC) of colistin against the isolates were lower than 2 μg/mL, and no resistance was seen. According to the DST results, isolates were categorized to 17 MDR, 13 mono‐drug resistance, and 10 susceptible isolates.

**TABLE 2:** Antibiotic susceptibility patterns of *P. aeruginosa* isolates.

Antibiotics	Sensitive *N* (%)	Intermediate *N* (%)	Resistant *N* (%)
Imipenem	22 (55)	0	18 (45)
Meropenem	24 (60)	0	16 (40)
Ciprofloxacin	27 (67.5)	0	13 (32.5)
Ceftazidime	24 (60)	0	16 (40)
Cefotaxime	24 (60)	0	16 (40)
Gentamicin	24 (60)	0	14 (35)
Piperacillin	24 (60)	0	16 (40)
Colistin	40 (100)	0	0 (0)

### 3.2 Phenotypic assessment of ESBL, metallo‐β‐lactamase, and carbapenemase

While the ESBL activity was not detected in any of the isolates, 12 (30%) isolates were positive for MBL, and 16 (40%) isolates had carbapenemase activity.

### 3.3 Biofilm formation

All the isolates (100%) were positive for biofilm production. Seventeen (42.5%) isolates were strong biofilm producers and 14 (35%) isolates were moderate producers. Moreover, biofilm production was weak in 9 (22.5%) isolates. The biofilm‐producer isolates had higher levels of antibiotic resistance (Table 3).

**TABLE 3:** Distribution of biofilm formation among *P. aeruginosa* isolates and correlation between biofilm production and antibiotic resistance patterns or co‐presence of virulence factors.

Biofilm production	Isolates *N* (%)	Antibiotic resistance phenotype			Number of virulence factors					
		MDR	Mono‐drug resistance	S	1	2	3	4	5	6
Strong N (%)	17 (42.5%)	11	4	2	0	2	5	3	3	4
Moderate N (%)	14 (35%)	5	6	3	1	3	1	1	5	3
Weak N (%)	9 (22.5%)	1	3	5	2	1	2	0	3	1
Total N (%)	40 (100%)	17 (42.5%)	13 (32.5%)	10 (25%)	3	6	8	4	11	8
Pearson Chi‐square *p*‐value		0.045			0.445					

*Note*: *p*‐value <0.05 considered as a significant correlation.

Abbreviations: MDR, Multi‐drug resistant; S, Susceptible phenotype.

### 3.4 ESBL and carbapenemase‐related genes

Among the ESBL genes, *bla*_TEM_, *bla*_CTX_, and *bla*_SHV_ genes were positive in 15 (37.5%), 8 (20%), and 6 (15%) isolates, respectively. MBL and Carbapenemase genes were less frequent and only *bla*_VIM_ gene was present in the isolates (30%), and *bla*_IMP_ and *bla*_NDM_ genes were not detected. Moreover, *bla*_OXA‐48_ and *bla*_OXA‐23_ genes were found in 7 (17.5%) and 1 (2.5%) isolates and no isolate possessed *bla*_OXA‐11_ gene (Table 4). The co‐occurrence of different types of β‐lactamase was seen in 15 isolates and the details are shown in Table 5.

**TABLE 4:** Distribution of ESBL, MBL, and carbapenemase genes among 40 *P. aeruginosa* isolates.

ESBL		*bla*_TEM_	*bla*_SHV_	*bla*_CTX‐M_
Genotypic	Phenotypic			
16 (40%)	0 (0%)	8 (20%)	6 (15%)	15 (37/5%)
MBL		*bla*_VIM_	*bla*_IMP_	*bla*_NDM_
Genotypic	Phenotypic			
12 (30%)	12 (30%)	0	0	12 (30%)
Carbapenemase		*bla*_OXA‐48_	*bla*_OXA‐23_	*bla*_OXA‐11_
Genotypic	Carba NP			
16 (40%)	16 (40%)	0	1 (2/5%)	7 (17/5%)

**TABLE 5:** The details of co‐presence of *bla* genes among *P. aeruginosa* isolates.

β‐lactamase class	Detection of genes by PCR assays	Isolates (*N*)	Phenotype
ESBL + MBL	(*bla*_TEM_ *, bla*_SHV_ *, bla*_CTX‐M_) + (*bla*_VIM_)	2	MDR
ESBL + MBL	(*bla*_TEM_) + (*bla*_VIM_)	3	MDR
ESBL + MBL	(*bla*_TEM_ *, bla*_SHV_) + (*bla*_VIM_)	1	MDR
ESBL + MBL	(*bla*_TEM_ *, bla*_CTX‐M_) + (*bla*_VIM_)	1	MDR
ESBL + Carbapenemase	(*bla*_TEM_) + (*bla*_oxa‐48_)	1	MDR
ESBL + Carbapenemase	(*bla*_TEM_ *, bla*_SHV_ *, bla*_CTX‐M_) + (*bla*_oxa‐23_)	1	MDR
ESBL + Carbapenemase	(*bla*_TEM_ *, bla*_SHV_) + (*bla*_oxa‐48_)	1	MDR
MBL + Carbapenemase	(*bla*_VIM_) + (*bla*_oxa‐48_)	1	MDR
ESBL + MBL + Carbapenemase	(*bla*_TEM_) + (*bla*_VIM_) + (*bla*_oxa‐48_)	2	MDR
ESBL + MBL + Carbapenemase	(*bla*_TEM_ *, bla*_CTX‐M_) + (*bla*_VIM_) + (*bla*_oxa‐48_)	1	MDR
ESBL + MBL + Carbapenemase	(*bla*_TEM_ *, bla*_SHV_ *, bla*_CTX‐M_) + (*bla*_VIM_) + (*bla*_oxa‐48_)	1	MDR
Total		15	

Abbreviation: MDR, Multi‐Drug Resistant.

### 3.5 Virulence factors

Among the virulence genes, *lasB* and *exoA* genes were detected in 38 (95%) isolates. The other genes including *plcH*, *exoS*, and *nan1* were present in 37 (92%), 36 (90%), and 16 (40%) isolates, respectively. Although we did not find any correlation between the virulence and β‐lactamase genes, the co‐existence of virulence genes (*lasB*, *exoA*, *plcH*, *exoS*, and *nan1*) was observed among the isolates. The *algD* gene was present in 17 (43%) isolates and all of them were strong biofilm producers.

### 3.6 ERIC‐PCR typing

**FIGURE 1:**
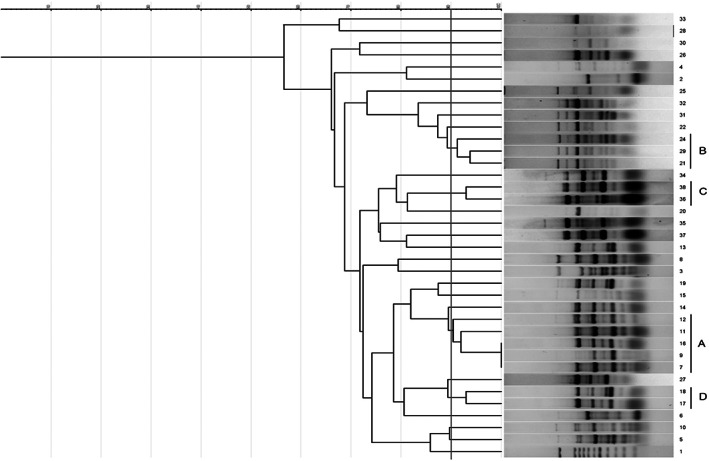
Dendrogram showing relatedness between ERIC‐PCR patterns of 36 *P. aeruginosa* isolates; Lane 1, 100 bp size marker; A–D, Four ERIC types.

ERIC‐PCR typing indicated high genetic diversity among the isolates. The results of genotyping showed that 36 isolates were classified into 28 ERIC types according to a 90% cut‐off (Figure 1). No band was detected following ERIC‐PCR in four isolates, and thereby they were non‐typeable. According to our analysis, 12 isolates were clustered in four main genotypes (A–D). The predominant type was type A, and it contained five isolates, followed by B (three), C (two), and D (two). Other 24 isolates possessed different banding patterns and they were distributed in 24 single types (Figure 1).

## 4 DISCUSSION

The growing rates of antibiotic resistance in *P. aeruginosa* neutralize antibiotic efficacy against infections caused by this opportunistic agent. In this study, we isolated *P. aeruginosa* from burn wounds and determined antibiotic resistance rate, biofilm production, and their virulence factors. Resistance to carbapenem antibiotics such as imipenem and meropenem was 45% and 40%, respectively. While these rates in a study conducted in Poland were 41% and 61.6%,^24^ and the International Nosocomial Infection Control Consortium (INICC) has reported a resistance rate of 47.2% for imipenem among clinical *P. aeruginosa* isolates, collected from different geographical regions, including Europe, Africa, Asia, and South America.^25^, ^26^, ^27^ In a previous study in the USA, meropenem resistance of 23.7% was reported among *P. aeruginosa* isolates.^28^

Interestingly, meropenem resistance has been demonstrated to be higher in *P. aeruginosa* isolates from cystic fibrosis patients.^29^ It is speculated that a complicated environment and a chronic infection in cystic fibrosis lungs are responsible for the higher resistance rates.^29^ Local studies from Iran have reported higher carbapenem resistance in *P. aeruginosa* isolates from burn wounds. Moreover, the imipenem resistance rate was found to be 58% and 94% in Shahrekord and Isfahan, respectively.^30^, ^31^

Also in a report from India, 61% of *P. aeruginosa* isolates from burn wounds were imipenem‐resistant.^11^ Altogether, it seems that the prevalence of carbapenem resistance depends on the geographical area of studies. MBL and carbapenemase enzymes are considered to be the main underlying carbapenem resistance. In the current study, 40% of isolates were positive for encoding at least one of the MBL or carbapenemase enzymes, and co‐existence of ESBL, MBL, and carbapenemase genes was observed in 37.5% of isolates. The co‐existence of these enzymes resulted in high levels of β‐lactam resistance, and as shown in Table 5, the co‐presence of these genes was related to the formation of the MDR phenotype. Also, *bla*_TEM_ (37.5%), *bla*_VIM_ (30%), and *bla*_CTX‐M_ (20%) were the most common β‐lactamase genes among the isolates. In other study by Peymani et al., the *bla*_TEM‐1_ (26.7%) and *bla*_CTX‐M‐15_ (17.3%), were the most common genes.^32^ The prevalence rate of ESBL in the study performed by Senthamaria et al.,^33^ Begum et al.,^34^ and Mirsalehian et al.,^35^ was 42.3%, 37.8%, and 39.4%, respectively.

In our study, all the isolates were biofilm producers, however, the intensity of biofilm was different among the isolates. In previous studies, 77.5%, 86.5%, and 100% of *P. aeruginosa* isolates were reported to be the biofilm producers, which supports our finding.^36^, ^37^ Similar to Ratajczak, et al.'s study, we found that the formation of biofilm is significantly stronger in MDR isolates, and 64.7% (11 out of 17 isolates) of strong biofilm producer isolates were MDR.^24^

The synergistic effect of antibiotic resistance and biofilm formation has been reported in *P. aeruginosa* and other bacterial pathogens, and several studies have displayed that biofilm formation is stronger in MDR strains of *P. aeruginosa*.^3^ While biofilm protects the bacterial cell from exposure to antibiotics and increases the probability of horizontal transfer of antibiotic‐resistance genes, antibiotic‐resistant bacteria form stronger biofilms. In addition, biofilm‐forming isolates have different MIC amounts than planktonic cells, and a combination of antibiotics probably contributes to the elimination of biofilm‐forming strains.^3^

In the present study, the most frequent virulence genes were *lasB* and *toxA*, which were present in 95% of isolates. In Ratajczak and colleagues' survey,^24^ *lasB* gene was present in 93.1% of *P. aeruginosa* clinical isolates, but this rate was estimated to be 86% and 75% in the other studies from China and India, respectively.^38^, ^39^ Although the rates reported by the above‐mentioned studies are lower than our results, elastase seems to be an important and frequent virulence factor of clinical *P. aeruginosa* isolates. We found that 95% and 90% of our isolates were positive for *toxA* and *toxS* genes. Both genes are common virulence factors among the *P. aeruginosa* isolates and other studies have also reported that most of the clinical and environmental isolates harbor virulence traits.^7^, ^40^ In other study conducted by Bogiel et al., PCR results indicated 58.9% and 96.3% of the isolates harbored *toxS* and *toxA* genes, respectively.^41^

Although Khosravi and colleagues^40^ have demonstrated that the existence of *toxA* and *toxS* genes is related to high antibiotic resistance in *P. aeruginosa* isolates, we did not find any significant correlation between the presence of these virulence factors and high antibiotic resistance rate.

The *plcH* gene is a source of hemolytic phospholipase C in *P. aeruginosa*.^42^ This virulence factor has a link to the high growth rate and pathogenicity, and mutant isolates have attenuated pathogenicity and slow growth rate.^42^ We found the *plcH* gene in 92.5% of the isolates, and this factor as well as *toxA*, *lasB*, and *toxS* could be related to the high pathogenicity of the studied isolates.

We investigated the genetic relatedness of the *P. aeruginosa* isolates using ERIC‐PCR fingerprinting, and the results showed high genetic diversity. Most carbapenem‐resistant isolates (12/16) were classified into four ERIC types (A‐D). ERIC patterns of other carbapenem‐resistant isolates were also comparable with type A (lanes no. 14 and 19) and type B (lanes no. 31 and 32) (Figure 1). It seems that these genotypes are circulating strains among hospitalized patients in various wards of the hospitals. Notable antimicrobial resistance and biofilm formation ability were identified in these types (Table 6), and these factors are associated with long‐term persistence in a medical setting.^43^, ^44^

**TABLE 6:** Characteristics of the predominant genotypes.

ERIC type (Included isolates)	Virulence genes	Resistance profile	Biofilm formation
A (5)	*toxA, toxS, lasB, plcH, algD*	MDR	Moderate
B (3)	*toxA, toxS, lasB, nan1, plcH, algD*	MDR	Strong

Abbreviation: MDR, Multi‐Drug Resistant.

According to the cut‐off, most of the isolates (*n* = 24) showed high‐level heterogeneity. These isolates, classified into 24 single types, were susceptible or did not show high‐level antimicrobial resistance. This diversity could be due to environmental or exogenous sources of the isolates. Based on the ERIC‐PCR method, four isolates were nontypeable; therefore, 90% (36/40) efficiency was calculated for this method in this study.

## 5 CONCLUSION

Antibiotic resistance of *P. aeruginosa* is considerable among the burn wound samples. Biofilm production is a synergistic factor that amplifies antibiotic resistance in these isolates, and alternative treatment for the elimination of biofilm could help decrease the antibiotic resistance rate in the life‐threatening burn infections by *P. aeruginosa*. Also, the high prevalence of virulence factors such as *toxA*, *plcH*, *toxS*, and *lasB* in our isolates shows that these factors are important in the pathogenesis of these bacteria in burn wounds. Innovation of new strategies for the inhibition of these virulence factors could be also beneficial for the treatment of burn infections by *P. aeruginosa*.

## AUTHOR CONTRIBUTIONS

SGH, HH, SKH, SGH, and MKZ substantially contributed to the conceptualization, methodology, validation, and investigation of the work. EK and HK have been involved in data curation, supervision, writing, and acquisition of data or revised the review article for intellectual content. All authors agreed and confirmed the manuscript for publication.

## CONFLICT OF INTEREST

The authors have no relevant affiliations or financial involvement with any organization or entity with a financial interest in or financial conflict with the subject matter or materials discussed in the manuscript. The authors report no conflict of interest in this study.

## Data Availability

The authors confirm that the data supporting the findings of this study are available within the article.
